# Effects of Nutrition Intervention on Blood Glucose, Body Composition, and Phase Angle in Obese and Overweight Patients with Diabetic Foot Ulcers

**DOI:** 10.3390/nu14173564

**Published:** 2022-08-30

**Authors:** Raedeh Basiri, Maria T. Spicer, Thomas Ledermann, Bahram H. Arjmandi

**Affiliations:** 1 Department of Nutrition and Food Studies, George Mason University, Fairfax, VA 22030, USA; 2 Institute for Biohealth Innovation, George Mason University, Fairfax, VA 22030, USA; 3 Department of Nutrition and Integrative Physiology, Florida State University, Tallahassee, FL 32306, USA; 4 Center for Advancing Exercise and Nutrition Research on Aging, Florida State University, Tallahassee, FL 32306, USA; 5 Department of Family and Child Sciences, Florida State University, Tallahassee, FL 32306, USA

**Keywords:** nutrition education, DFU, nutrition supplementation, body composition, phase angle, protein, macronutrients, micronutrients, chronic wounds, wound healing, diabetic foot ulcer

## Abstract

Nutrition can play an important role in the treatment of chronic wounds such as diabetic foot ulcers (DFUs); however, diet therapy is not currently part of the standard care for DFUs. There are numerous controversies about dietary recommendations, especially regarding calories and macronutrients, for overweight and obese patients with DFUs. This study examined the effects of nutrition education and supplementation on body composition in overweight and obese patients with DFUs. Twenty-nine patients with DFUs between the ages of 30 and 70 years were randomly assigned to either the treatment group (nutritional supplements, diet education, and standard care) or the control group (standard care). At baseline, the mean body mass index (BMI) was 33.5 kg/m^2^ for the treatment group and 34.1 kg/m^2^ for the control group. HbA1c decreased in both groups, with no significant difference between the groups. On average, patients in the treatment group lost less lean body mass and gained less fat than the control group ((3.8 kg vs. 4.9 kg) and (0.9 kg vs. 3.6 kg), respectively). While the interaction between group and time did not reach statistical significance for any of the study variables after adjustments for confounding variables, the observed changes are clinically relevant.

## 1. Introduction

Diabetic foot ulcers (DFUs) are among the most common complications of uncontrolled diabetes [1]. It has been reported that 25% of patients with diabetes develop DFUs during their lifetime [2]. DFUs significantly affect the patient’s health and socioeconomic well-being and negatively affect the quality of life of the patients and their family members [3]. Nutrition can play a key role in the prevention and improvement of the clinical outcomes of DFUs [4]. In patients with chronic wounds, cellular activity and inflammation in the healing wound increase metabolic needs; therefore, they require more energy and a higher nutrient intake. In DFUs, the hypermetabolism nature of the wound, as well as a decreased sensitivity to insulin, increased counter-regulatory hormones such as cortisol, catecholamines, and glucagon due to a high level of stress, and not enough energy intake results in the body utilizing muscle proteins as a source of energy [5,6]. Apart from the wound itself, the kinetics of whole-body protein metabolism are elevated in diabetes and the net balance is diminished [7]. Increased protein catabolism and negative nitrogen balance have been reported in patients with uncontrolled diabetes [8]. Additionally, it has been reported that energy expenditure is significantly higher in patients with type 2 diabetes, in comparison with non-diabetic individuals [9,10]. Despite the higher need for energy sources and essential nutrients during wound healing, diabetic patients are usually recommended to follow low-calorie/low-carbohydrate diets to better manage their glycemic indices and related complications, particularly if they are overweight or obese [11]. However, restrictive diets could result in an inadequate intake of essential micronutrients, as well as the energy sources and protein that are vital for wound healing. It has been reported that patients with DFUs have a significantly lower intake of energy, protein, and micronutrients compared to dietary reference intakes (DRI), which are designed for a healthy population [12,13,14]. A significantly low dietary intake of energy and nutrients has also been shown in overweight and obese patients with DFUs [15]. Insufficient energy intake can result in muscle wasting, the loss of subcutaneous tissue, and consequently, poor wound healing [16]. Protein is responsible for cell proliferation, collagen, and connective tissue synthesis, as well as the antibody synthesis needed for immune system function [17]; therefore, adequate protein intake supplemented by non-protein energy sources promotes a positive nitrogen balance, which is crucial for improving wound healing in DFUs. Although an increased need for energy and nutrient intake has been established in patients with chronic wounds [18] and an inadequate intake of energy sources and nutrients has repeatedly been reported in patients with DFUs, there are still controversies about the nutritional recommendations for DFU patients, particularly for those who are overweight or obese. This study examined the effects of nutrition education and supplementation on long-term blood glucose control, body composition, and phase angle as an indicator of cellular health and cell membrane integrity in patients with DFUs.

## 2. Materials and Methods

### 2.1. Screening and Recruitment

This study was approved by the Institutional Review Board (IRB) of Tallahassee Memorial HealthCare (TMH, Tallahassee, FL, USA) and Florida State University, and is registered at clinicaltrials.gov NCT04055064. The study was advertised at the Tallahassee Memorial Wound Healing Center; interested participants were prescreened by one of the medical staff or a nurse at the clinic, based on the inclusion/exclusion criteria. The potential participants were then scheduled for a screening visit with the researcher. Patients were included in the study if they were between the ages of 30 and 70 years, had a body mass index (BMI) ≥ 25 kg/m^2^, had at least one diabetic foot ulcer of grade 1A [19], and were receiving medications for glycemic control.

Patients were excluded from the study if they were pregnant or lactating, had used bioengineered tissue within the four weeks before baseline, had high concentrations of hemoglobin A1C (HbA1c) of > 12%, known immunosuppression, liver failure/cirrhosis, active malignancy, myocardial infarction or heart failure in the past three months, chronic kidney disease, underwent radiation therapy for the treatment of their wounds, excessive use of alcohol according to the standards of the World Health Organization, or were subject to any physiological or mental condition that might affect the study regimen.

After screening, eligible patients were provided with details of the study and were asked to sign the consent form if they were interested in participating in the study. Participants were then randomly assigned to either the control or the treatment group.

### 2.2. Study Intervention

Standard wound care was provided to all the participants at the TMH Wound Healing Center. Additionally, patients in the treatment group were asked to consume more low-fat protein sources with high bioavailability, vegetables, high-fiber carbohydrates, and a lower amount of simple carbohydrates. Participants were also educated about the different food groups and were given examples of healthier food items in each group. The nutrition education was conducted by the researcher (nutritionist) for at least 10 min at baseline and was then repeated every four weeks for each patient in the treatment group. The treatment group was also provided with two servings of Boost Glucose Control nutritional formula (Nestlé Health Science, NJ, USA) and instructed to consume one supplement in the morning and one in the afternoon, during the study. Consuming two servings of supplements provided patients with extra energy, protein, and essential vitamins and minerals. Table 1 shows the energy and macronutrient contents of the supplement. The complete nutrient content of the supplement has been published elsewhere [15].

The supplement used in this study was designed for diabetic patients and contained a slow-release carbohydrate source called tapioca dextrin, which is digested slowly. Tapioca is resistant to amylase and prevents a sudden increase in blood glucose [20]. This study aimed to provide patients with adequate supplements that could help the patients to meet their extra need for energy and protein and to support them with at least 50% of the Recommended Dietary Allowance (RDA) recommendations for the essential vitamins and minerals for wound healing. We anticipated that the combination of nutrition education and supplements could significantly improve the dietary intake of participants and support them in meeting their nutrient recommendations.

### 2.3. Anthropometric and Body Composition Measurement

Height was self-reported, and weight was measured at baseline and every four weeks during the study, using a stand-on scale (Seca Mechanical Column Scale; Hamburg, Germany). BMI was calculated from weight and height using the BMI = weight (kg)/Height^2^ (m^2^) formula. Body composition was evaluated using a bioelectrical impedance analyzer (BIA) 310 e (Biodynamics Corporation, Seattle, WA, USA) which has been shown to accurately estimate body cell mass and lean body mass [21]. The within-day and between-day coefficients of variation (CVs)% for hand-to-foot (whole-body) model impedance have been reported as 0.2% to 0.7% and 0.9% to 1.8%, respectively [22]. Patients were instructed to remove their right shoe and sock and lay down on their backs. Their feet were 12 to 18 inches apart, while their hands were placed palm-down at 6 to 12 inches from the torso. Two adhesive sensor pads were placed on the right wrist/hand and two on the right ankle/foot. A small current (800 µA at 50 kHz) was passed through the electrodes to measure fat body weight (kg), lean body mass weight (kg), and reactance and resistance; the measurements were used for calculating the phase angle (PA). Fat body weight is the total amount of stored lipids in the body and consists of subcutaneous and visceral fat. Lean body mass weight was calculated by subtracting body fat (kg) from total body weight (kg). PA indicates a relationship between electric resistance (R) and reactance (Rc) and is an indicator of cellular health and function [15,16]. Lower phase angles are correlated with the duration of disease, inflammation, malnutrition, and mortality in diabetic patients [23,24,25,26]. PA was calculated directly from reactance and resistance, using the PA = arctangent reactance (ohm)/resistance (ohm) × 180°/π formula [27].

### 2.4. HbA1c Measurement

To assess the effects of the intervention on long-term glucose homeostasis, HbA1c was evaluated at baseline and the end of the study, using the HbA1c Now+ test (Polymer Technology Systems, Indianapolis, IN, USA).

### 2.5. Dietary Assessment

The dietary intake of participants was estimated using 24-hour recall forms. Participants were asked about all the foods and beverages consumed during the last 24 h as well as any prescribed or voluntary use of nutritional supplements. Nutrient intake was estimated using the Food Processor SQL, version 11.1.480 (ESHA’s Food Processor^®^, Salem, OR, USA).

### 2.6. Statistical Analysis

The Statistical Package for Social Science (SPSS), version 25.0 (SPSS, Inc., Chicago, IL, USA), was utilized to analyze our data. For all tests, *p* < 0.05 was set as the statistically significant level. Population characteristics were evaluated at baseline using descriptive statistics. An independent sample *t*-test was used to compare the means of potential confounding variables between groups at baseline; if the effect was significant, they were set as covariates in the model. The independent sample *t*-test was also used for the comparison of HbA1c concentrations between groups at baseline and the end of the study. All the other variables were analyzed using multilevel modeling (mixed model), while Bonferroni’s post hoc test was utilized for pairwise comparisons if the F-statistic was significant.

## 3. Results

Out of 95 patients who were screened for the study, 42 met the inclusion criteria and were interested in participating in this study. Thirteen patients were then excluded from the study, due to a change in their clinic or because of missing their next two appointments. Laboratory, clinical, and statistical analyses were performed for a total of 29 patients.

### 3.1. General Characteristics

Descriptive data of the relevant characteristics of the participants at baseline are outlined in Table 2. There were no statistically significant differences in ethnicity, age, BMI, HbA1C, duration of diabetes, wound area, or wound age estimation among the participants of the two groups. Gender distribution differed in the groups; however, the effects of gender on each variable were evaluated and, if the effect was significant, it was added to the model as a covariate. Participants in the treatment group had a longer duration of diabetes, in comparison with the ones in the control group (14.4 ± 8 years vs. 11.7 ± 6 years, respectively), but this difference did not reach statistical significance. No significant differences were observed between the two groups in terms of the indicators of socioeconomic status (SES) and other factors that could affect the nutritional status of patients, such as appetite problems, previous unintentional weight loss, and cultural and religious dietary restrictions. Living alone, having financial concerns, being employed, and having food needs were considered indicators of SES. Referrals to registered dietitians (RDs) were not part of the standard care for patients with DFUs and only 38% of the patients had visited RDs at least once in the past.

### 3.2. Dietary Intake of Participants at Baseline and during the Follow-Up

The mean dietary intake of participants in terms of energy and protein was 50% and 48.7% of the recommendations when compared to the minimum recommendations for energy (30 kcal/kg) and protein (1.2 g/kg) made by the National Pressure Ulcer Advisory Panel (NPUAP). The mean dietary intake of essential micronutrients for wound healing was also alarmingly lower than DRI in this population. Details about the change in energy, protein, and micronutrient intake of the treatment and control group during the study have been published elsewhere [15]. In summary, the dietary intake of energy did not change significantly during the study for either the treatment or control group, and the interaction between time and group was not statistically significant. Compared to NPUAP recommendations, energy intake increased from 52.0% to 68.0% in the treatment group and from 43.7% to 57.8% in the control group. The increase in the protein intake of the treatment group was higher (from 54.5% to 84.9%) than the control group (from 43.1% to 54.7%) when it was compared with NPUAP recommendations. The interaction between group and time was not statistically significant; however, the change in dietary intake of protein in the treatment group is clinically relevant. Although the treatment group was provided with an extra 500 kcal of energy and an additional 28 g of protein, they still could not meet the NPUAP recommendations for energy and protein intake. The dietary intake of copper, zinc, vitamin A, vitamin C, and vitamin E significantly increased in the treatment group; however, no significant changes in the dietary intake of the participants in the control group were observed during the follow-up.

### 3.3. Hemoglobin A1c

The concentration of HbA1c decreased at a similar rate in both the treatment and control groups (0.31% and 0.39%, respectively). At the end of the study, there were no significant differences between the concentration of HbA1c among the groups; therefore, supplementation with extra energy sources and nutrients did not have any negative effect on long-term blood glucose control in patients with DFUs.

### 3.4. Anthropometrics and Body Composition

#### 3.4.1. Changes in Body Mass Index

The mean BMI at baseline for patients in the treatment and control groups were 33.5 kg/m^2^ and 34.1 kg/m^2^, respectively. We examined the potential effects of gender and age on BMI; however, since their effects were not significant, we did not include them in the statistical model. The interaction between time and group did not reach statistical significance for BMI. Therefore, the intervention did not have any negative effects on the BMI of the patients in the treatment group.

#### 3.4.2. Changes in Lean Body Mass

The mean LBM of the treatment group was slightly lower than that of the control group (68.2 kg vs. 69.1 kg, respectively) at baseline; however, the difference was not statistically significant. The effects of gender (*p* < 0.001), age (*p* = 0.001), wound age estimation (*p* < 0.001), and duration of diabetes (*p* < 0.001) on the LBM change were significant; therefore, we added these factors as covariates to the statistical model. Although the mean LBM of both groups was lowered during the study, patients in the treatment group lost less LBM than the patients in the control group (3.8 kg vs. 4.9 kg, respectively). The interaction between time and group did not reach statistical significance for LBM after adjustments for the confounding variables; however, the difference is clinically relevant.

#### 3.4.3. Changes in Body Fat Weight

At baseline, there was no significant difference in body fat weight between the two groups. We examined the potential effects of confounding variables, such as gender (*p* = 0.04), duration of diabetes (*p* < 0.001), and wound age estimation (*p* < 0.001). All the significant confounding variables were included as covariates in the model. The body fat weight changed in the treatment group from 31.7 kg to 32.1 kg and in the control group from 34.3 kg to 35.9 kg. While the interaction between time and group did not reach statistical significance for body fat weight after adjustments for confounding variables, these changes are clinically relevant.

### 3.5. Changes in Phase Angle

The PA for the participants in the treatment group was slightly higher than that in the control group at baseline; however, the difference was not statistically significant (7.0° vs. 6.8°, respectively). We examined the potential effects of the confounding variables age, gender, BMI, duration of diabetes, and wound age estimation. Age was the only significant variable (*p* = 0.001); therefore, it was kept in the model as a covariate. After adjustments for the effects of age, PA was decreased by 0.3° in the treatment group and 0.6° in the control group during the study period. Even though the difference between the two groups did not reach statical significance (*p* = 0.09) for PA, these changes are clinically relevant.

## 4. Discussion

The findings of this study showed that supplementing the diet with extra energy sources and nutrients did not have any negative effects on long-term blood glucose control or the body composition of overweight and obese patients with DFUs when combined with nutrition education. Additionally, our results showed that our intervention had some positive effects on the body composition and PA of DFU patients in the treatment group. As is similar to the findings in other research [16,28,29,30], LBM decreased in our participants during the study; however, our treatment group could maintain LBM better than the control group. Although the effect was not statistically significant, preventing the loss of LBM leads to a better nitrogen balance, which is important for the faster healing of chronic wounds [4,28,31,32]. This could also be one of the reasons that DFUs in our treatment group healed 12.85-fold faster than in the control group [15].

While we had no intentions to decrease body fat during the healing time, this might have happened as a result of educating our treatment group about consuming nutrient-dense foods. The observed increase in protein intake along with receiving an adequate quantity of micronutrients [12] could have positive effects on regulating patients’ appetite and decreasing cravings for sugar and sweets in the treatment group. This could be confirmed by our data, which showed lower sugar intake and no significant increase in energy intake in the treatment group during the study period, despite their receiving extra energy (500 kcal/d) through supplements. Our results showed that the mean sugar intake decreased by 20 g/d in the treatment group and 8 g/d in the control group. This highlights the importance of nutrition education in changing eating behaviors in this population.

We previously showed that our intervention also significantly decreased inflammation in the treatment group [33]. Rieu et al. [34] reported that the reduction of low-grade inflammation (lower IL6 and IL1β) decreased muscle mass loss and increased muscle protein synthesis by 24.8% (*p* < 0.05). Therefore, the better preservation of lean body mass that occurred in the treatment group might have happened partly as a result of decreased inflammation in the treatment group, due to an increased intake of micronutrients and antioxidants. Nonetheless, further studies are needed to replicate our findings.

Although the change in PA did not reach statistical significance for the duration of the study, scientific evidence has shown that one grade increase in PA is associated with a 33% reduction in the risk of mortality or renal death in diabetic patients [35]; therefore, the observed lower decrease in PA in the treatment group relative to the control group is clinically relevant. Our results showed that HbA1c decreased in both groups during the study and there were no significant differences between the two groups during the study. Therefore, supplementing DFU patients with additional energy sources and nutrients did not have negative effects on long-term glucose control when it was combined with nutrition education. This confirms that overweight and obese patients with DFUs could benefit from a generalized diet when it is combined with nutrition education. Nutrition education for DFU patients should prioritize dietary needs for wound healing. Consuming nutrient-dense foods should be emphasized for meeting energy and nutrient needs during wound healing. When the wound is fully healed, calorie restrictions with better nutritional support may be applied if necessary. Other studies should be conducted using a larger population, applying a similar approach, to confirm our results. If our observations are confirmed in similar studies, our method would add new approaches to the treatment of patients with DFUs.

To our knowledge, this is the first study that evaluates the effects of nutrition education and supplementation with extra calories, protein, and micronutrients on long-term blood glucose control and body composition in overweight and obese patients with DFUs. The strength of our study was that the patients were educated to choose nutrient-dense foods in addition to receiving supplements, which made it easier for them to meet their dietary requirements. It is also important to note that the supplement was tolerated well, and participants did not report any adverse effects related to the use of the supplement. One of the limitations of this study was that due to the small population in the area, the effects of nutrition education or supplementation on the outcome variables could not be evaluated independently. Additionally, the participants of this study were not provided with individualized dietary recommendations. Different medications might have different effects on blood glucose concentrations and wound outcomes; however, we did not collect data about the medications used by our participants. Currently, there are no recommendations regarding the dietary intake of energy sources and protein for patients with DFUs; therefore, the assessment of the dietary intake of our participants was conducted based on the recommendations from NPUAP, which are for patients with pressure ulcers. The supplement used in this study was not designed for patients with DFUs; however, it was the most appropriate manufactured formula that could help our participants to meet most of their dietary needs. Future research should identify the optimum amounts of energy sources and nutrients for faster wound healing in diabetic patients with foot ulcers. Routine visits with a dietitian are essential for assessing the dietary needs of patients and designing individualized nutrition therapy, which can result in effective clinical outcomes. Identifying the adequate dietary intake of macro- and micronutrients in diabetic patients with foot ulcers, especially for those who are overweight or obese, is critical for expediting the wound-healing process and can make a substantial difference to medical expenses and quality of life in this population.

## 5. Conclusions

Dietary recommendations for overweight and obese individuals with DFUs should prioritize proper wound healing by recommending that patients consume adequate energy sources and essential nutrients.

## Figures and Tables

**Table 1:** Energy and macronutrient contents of one serving (237 mL) of the nutritional supplement.

Nutrient	Amount
Calories (kcal)	250
Calories from Fat (kcal)	110
Total Fat (g)	12
Saturated Fat (g)	1.5
Total Carbohydrate (g)	23
Dietary Fiber (g)	3
Sugars (g)	6
Protein ^1^ (g)	14

^1^ Includes protein from caseinate and L-arginine.

**Table 2:** Baseline characteristics of participants, according to group.

Variable	Treatment (*n* = 15)	Control (*n* = 14)	*p*-Value
Women/men	7/8	3/11	0.08
Age (year)			
Means ± SD	52.9 ± 9.74	53.8 ± 12.8	0.84
Ethnicity			
African American/white	4/11	3/11	0.75
BMI ^1^ (kg/m^2^)			
Means ± SD	33.5 ± 7.98	34.1 ± 6.04	0.84
HbA1C ^2^			
Means ± SD	7.95 ± 2.06	8.40 ± 2.16	0.57
Duration of diabetes			
Means ± SD	14.40 ± 8.03	11.7 ± 6.17	0.32
Wound age (months)			
Means ± SD	10.97 ± 15.09	10.58 ± 18.27	0.95
Smoking			
(yes/no)	3/12	3/11	1.00

^1^ BMI: body mass index. ^2^ HbA1C: Hemoglobin A1C.

## Data Availability

The datasets generated from this study are available from the corresponding author upon reasonable request.
